# The Effects of Chitosan on the Healing Process of Oral Mucosa: An Observational Cohort Feasibility Split-Mouth Study

**DOI:** 10.3390/nano13040706

**Published:** 2023-02-12

**Authors:** Gonçalo de Jesus, Lara Marques, Nuno Vale, Rui Amaral Mendes

**Affiliations:** 1 Centro Regional das Beiras, Universidade Católica Portuguesa, Estrada da Circunvalação, 3504-505 Viseu, Portugal; 2 OncoPharma Research Group, Center for Health Technology and Services Research (CINTESIS), Rua Doutor Plácido da Costa, 4200-450 Porto, Portugal; 3 CINTESIS@RISE, Faculty of Medicine, University of Porto, Alameda Professor Hernâni Monteiro, 4200-319 Porto, Portugal; 4 Department of Community Medicine, Information and Health Decision Sciences (MEDCIDS), Faculty of Medicine, University of Porto, Rua Doutor Plácido da Costa, 4200-450 Porto, Portugal; 5 Department of Oral and Maxillofacial Medicine and Diagnostic Sciences, Case Western Reserve University, 10900 Euclid Ave, Cleveland, OH 44106-7401, USA

**Keywords:** chitosan, wound healing, oral mucosa, oral surgery

## Abstract

The healing process is a dynamic process accompanied by some classical symptoms of inflammation such as redness, swelling, pain, and loss of function. Chitosan is a natural polymer with properties that contribute to tissue healing, with properties that could be applied in periodontal therapy, such as the wound healing of oral mucosa. This experimental split-mouth study aims to assess the possibilities of chitosan influencing the healing process of oral mucosa in eight patients, where the studied group was subjected to two oral surgeries: one with chitosan hydrogel into the socket and other without the biomaterial. A semi-quantitative analysis of the data was performed. Some classic signs of inflammation in a short period of time were observed where chitosan acted, compared to the control. An absence of bleeding was observed in the chitosan cases. According to the literature, chitosan recruits and activates neutrophils and macrophages and stimulates angiogenesis. Hemostatic and antimicrobial activity of chitosan also play an important role in wound healing. Chitosan seems to improve the postoperative quality of patients, allowing rapid wound healing with less complications.

## 1. Introduction

### 1.1. Wound Healing

**Figure 1:**
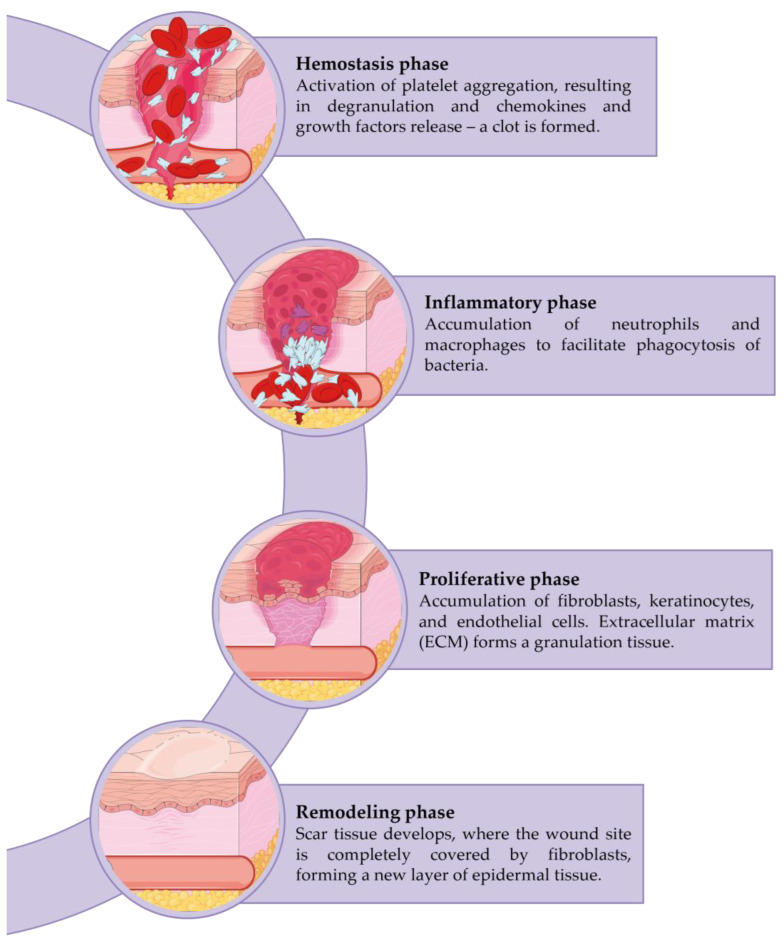
Phases of the wound healing process. Created with SMART—Servier Medical ART. Available online: https://smart.servier.com (accessed on 5 December 2022).

Wound healing is a dynamic and complex process initiated when tissue integrity is compromised. It is characterized by a sequence of orchestrated phases (Figure 1): hemostasis, inflammation, cellular migration, and proliferation, protein synthesis, wound contraction, and remodeling, which involves the recruitment of several soluble mediators, extracellular matrix (ECM) components, and parenchymal cells [1,2,3,4,5,6,7,8]. However, only three phases are distinguished: inflammation, proliferation, and remodeling, since an overlap of phases usually occurs [8,9,10].

Hemostasis and coagulation, are the first steps in the healing process and serve as the impetus for the subsequent phases [11,12,13]. Immediately after injury, the neuronal reflex mechanism occurs, which leads to the rapid contraction of vascular smooth muscle to prevent the extravasation of blood [13]. When a macrodisruption of the blood vessels occurs, blood spills into the injured area and contacts exposed collagen and other ECM components. This culminates in the release of clotting factors from the platelets and the formation of a blood clot. Thus, in addition to preventing the collapse of the vascular system, these phenomena prepare the injured tissue for the following phases. The clot is composed of ECM proteins (fibronectin, fibrin, vitronectin, etc.), which allow the subsequent establishment of the invading cells. Further, it also acts as a reservoir of growth factors required for the later phases. The clot is also considered a physical barrier against microorganisms [1,3,14,15]. 

Inflammation follows, with the invasion of neutrophils and macrophages attempting to remove damaged tissue and bacteria. This stage is commonly accompanied by some classical symptoms such as pain, redness, and edema [16]. Neutrophils are the first cells attracted by chemoattractive agents. Their major purpose is to phagocytize foreign and undesirable elements. They also produce a variety of proteins and reactive oxygen species (ROS) that complement their primary function [13,17]. Macrophages have a longer lifespan than neutrophils and play a significant role in the final stages of the inflammatory response, activating keratinocytes, fibroblasts, and endothelial cells, and providing an abundant reservoir of potent tissue growth factors [13].

The proliferative phase begins after several hours of injury. The migration and proliferation of epithelial cells occur during this phase, extending up to 5–14 days following tissue damage. This includes new tissue formation, fibroblast migration, and deposition of newly synthesized extracellular matrix (which replaces the provisional network composed of fibrin and fibronectin), granulation, and re-epithelialization, as well as the re-establishment of the vascular network. Myofibroblasts provide contractile forces that allow the wound edges to bind together [7,13,18].

Finally, the remodeling phase is characterized by a rearrangement and contraction of the newly formed matrix [8]. Over time, metabolic activity at the wound site decreases along with the growth arrest of capillaries and the decline of blood. The final result is a fully matured scar with a decreased number of cells and blood vessels and high tensile strength. The tissue-acquired final strength depends on the location of the repair and its duration, although it will never be the same as unwounded tissue, since collagen fibers may regain only up to approximately 80% of their original strength [13].

### 1.2. Chitosan

**Figure 2:**
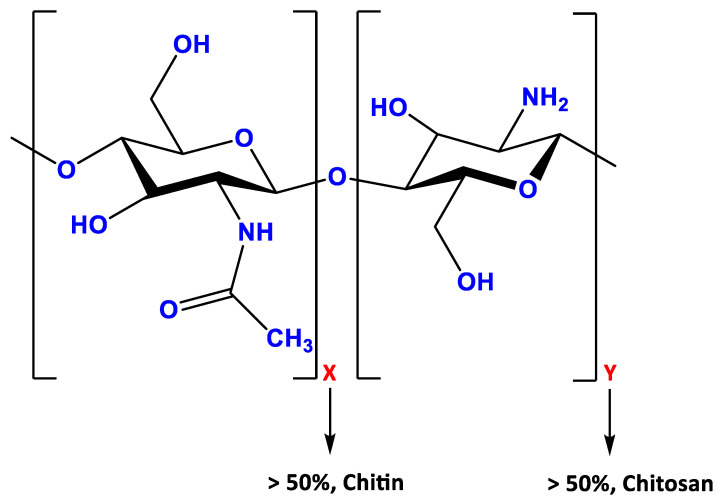
Chemical structure of chitosan. X and Y indicates the glucosamine and acetyglucosamine polymer, respectively.

Chitosan, a linear biopolymer extracted from N-acetylated chitin, is a cationic polysaccharide containing copolymers of glucosamine. Its molecular structure (Figure 2) comprises a linear backbone linked through glycosidic bonds with a random copolymer of β-(1–4)-linked D-glucosamine and N-acetyl-D-glucosamine [19,20,21,22]. Although it is a weak base insoluble in water, it becomes soluble in aqueous acidic media [21,23]. Its insolubility in neutral or basic solutions occurs due to its slightly crystalline character. However, an acidic environment enables the free amino groups of chitosan to become protonated. The high positive charge of chitosan allows the formation of a polyelectrolyte complex hydrogel with polyanionic species in an acidic environment [24].

The positive charge of chitosan usually provides a great interaction with cell membranes (negatively charged) due to ionic interchanges between the intercellular and extracellular medium. This means that highly deacetylated chitosan (with more positively charged amino groups) has a naturally strong tendency for cellular interaction [25].

Tissue engineering has shown interest in chitosan to culture hepatocytes, fibroblasts, and cartilage cells due to its scalloped structure and the ability to promote cell attachment and growth [26]. 

### 1.3. Properties

Chitosan displays a wide variety of applications as a biomaterial due to its excellent properties, such as antimicrobial activity, excellent behavior in the human body, its hemostatic effect, and its ability to adhere to different types of cells [27]. The effects of chitosan have been the subject of several toxicological studies in the medical field and, in general, its biocompatibility is demonstrated [20]. In addition, chitosan has been reported to be safe, since human volunteers were given daily oral chitosan levels of 6.75 g with no adverse effects [28,29].

Concerning periodontal tissue regeneration, chitosan membrane has been tested [30,31,32] and shown to be non-toxic, with no evidence of an inflammatory reaction [20]. The scientific community has long debated whether patients with allergies to marine crustaceans should avoid crustacean-derived biomaterials, since there is a portion of the human population, albeit small, that exhibits such allergies. It was then demonstrated that this allergy is caused by immunoglobulin E (IgE) antibodies to antigens present in the crustacean flesh and not in the shell [33], and therefore chitosan and chitin-derived products have been proven safe and well-tolerated in patients with a crustacean allergy [34]. No data on the carcinogenic potential of chitosan were found in the literature [35].

Chitosan has been reported to be highly biodegradable owing to the fact that its molecular chains can be degraded by enzymes such as lysozyme, chitinase, N-acetyl-D-glucosaminidase, and lipases under physiological conditions. The products of its degradation do not raise any critical concern because these products are oligosaccharides that are either incorporated into glycosaminoglycan and glycoprotein metabolic pathways or easily excreted in urine directly [7,25,29]. Chitosan can be degraded by lysozyme through the hydrolysis of acetylated residues, and the degradation products are non-toxic [29,36].

Many studies have evaluated the degradation rate of chitosan preparations by different methods and conclude that the degradation rate depends on their molecular weight and their preparation methods. For example, in vivo studies in rats revealed that degradation is less rapid as the degree of deacetylation becomes higher, which leads us to conclude that the degradation rate is inversely proportional to the degree of deacetylation [29,33].

Furthermore, chitosan has also been used in wound treatment, particularly in periodontal therapy for tissue reconstruction purposes, as it has minimal body reaction and the potential to promote cell proliferation of human gingival fibroblasts and stimulate wound healing. This biopolymer has been reported to play an important role in the early phase of wound healing because it increases the infiltration of polymorphonuclear cells (PMNs). It stimulates macrophages and mononuclear cells, as well as the synthesis of various growth factors that enhance wound healing and bone formation [24,37].

Chitosan also has an impact on the phases of cytokine production, giant cell migration, and the stimulation of type IV collagen synthesis. The stimulation of fibroblasts ensures the production of interleukin-8 (IL-8), acting actively in chemotaxis and angiogenesis. It performs complement activation in an alternative manner since it promotes the production of C5a, which in turn increases the migration and adherence of neutrophils and monocytes to vessel walls [38]. Chitosan can foster adequate granulation tissue formation accompanied by angiogenesis and regular deposition of collagen fibers [7]. It promotes granulation and organization, both of which are beneficial for the wound healing process [7].

Another remarkable characteristic is its antibacterial activity. Several studies documented the bacterial effectiveness of chitosan on Gram-negative and Gram-positive bacteria. Oral bacteria include Streptococcus, Staphylococcus, Actinobacillus, and various anaerobes, in particular, Bacteroides [20,23]. 

A key feature of chitosan, which differs from other polysaccharoses, is that it can form a polycationic structure by binding to anions on the bacterial surface. This finding allowed researchers to understand that the interaction between the anionic components of Gram-negative bacteria and the positive charge of chitosan can weaken the outer membrane barrier function of microorganisms, explaining its antibacterial effect [20].

Goy et al. [23] proposed three mechanisms of interaction between chitosan and microorganisms: a) the ionic surface interaction resulting in a wall cell leakage, b) the inhibition of the mRNA and protein synthesis via the penetration of chitosan into the nuclei of the microorganisms, and c) the formation of an external barrier chelating metal and provoking the suppression of essential-to-microbial growth. These mechanisms can occur simultaneously but with different intensities [23].

The effect of chitosan on *Actinobacillus actinomycetemcomitans* and *Streptococcus mutans* was evaluated in vitro and the results showed an antimicrobial action at a very low concentration. Moreover, chitosan selectively inhibited *S. mutans* adsorption to hydroxyapatite. Chitosan can also penetrate biofilms formed by the pathogenic fungus *Cryptococcus neoformans* and damage fungal cells, resulting in a reduction in the metabolic activity of the biofilms and cell viability [20].

This crustacean-derived biopolymer has also been reported to be a hemostatic agent. Clot formation appears to be due to an interaction between the cell membrane of erythrocytes and amino groups of chitosan, in absence of coagulation factors or platelets. As aforementioned, the difference between the charges of the biopolymer and the cell membranes again impacts the properties: the positive charge of chitosan and negative charge of the membranes of erythrocytes and platelets attract each other, leading to platelet activation and thrombus formation [29,39,40]. For instance, Celox®, HemCon®, and Quikclot® are chitosan-containing medical devices marketed in Europe and in the United States (US) for the treatment of bleeding and have been supported by evidence of reduced bleeding and enhanced hemostasis in experimental models [29].

Finally, the adhesion capacity of chitosan hydrogel has also been recognized. Its high adhesive force led by adequate water absorption capacity with the cationic nature promotes its binding to the negative mucosal surface. It suggests another important property: the intrinsic bioadhesive propriety of chitosan hydrogel that maintains an intimate contact with oral mucosa [26]. The variety of forms of chitosan in terms of molecular weight and degree of deacetylation affect solubility and mucoadhesivity. If the deacetylation degree decreases, the solubilization becomes more difficult and the mucoadhesive capacity of the polymer also decreases due to less positively charged amino groups available for the interaction with negatively charged residues of the mucus. This means that highly deacetylated chitosan has more mucoadhesion, which translates into longer retention at the site of action or absorption [25].

This experimental pilot study aims to assess the potential clinical impact of chitosan in the healing process of the oral mucosa, which may render it a subsequent application either after intra-oral surgical procedures (e.g., extraction of impacted teeth and biopsies), as well as a pharmacological adjunct in terms of the management of oral mucosa impairment related to oncological treatment (e.g., mucositis).

## 2. Materials and Methods

This observational cohort feasibility split-mouth study was approved by the Ethics Committee of Universidade Católica Portuguesa. All participants signed an informed consent in writing prior to the research being conducted. This study was conducted among patients requiring bilateral extraction of impacted mandibular third molars with similar Pell and Gregory classifications, where chitosan hydrogel was applied to the socket, to be later compared with the oral mucosa healing in a contralateral tooth extraction, where chitosan was not applied to the socket. In other words, each patient is simultaneously part of the experimental group (the chitosan sockets) and the control group (the sockets not treated with chitosan). All surgeries were performed by the same oral surgeon. Study randomization was ensured during surgery, which was performed without the patients knowing which socket the chitosan hydrogel was applied to.

The patients were selected according to the following inclusion criteria: (1) patients with indications for extraction; (2) submucous or impacted mandibular teeth suitable for performing a primary wound closure; (3) patients whose contralateral tooth is indicated for extraction; (4) patients without systemic diseases; and (5) patients who signed informed consent before conducting the healthcare intervention. In turn, exclusion criteria include: (1) teeth with indication for extraction but where primary wound closure could not be performed; (2) maxillary teeth indicated for extraction; (3) patients with systemic diseases; (4) pregnant and lactating patients; and (5) patients who could attend controls.

### 2.1. Preparation of the Hydrogel Chitosan

Chitosan granules lack sufficient tissue biocompatibility, which results in challenging regular epithelization [41]; therefore, the dissolution of chitosan was carefully performed. Hence, a chitosan hydrogel was prepared by dissolving chitosan (MW: 10–50 Da) (Sigma-Aldrich Co., Steinheim, Germany) in 1% acetic acid (Sigma-Aldrich Co., Steinheim, Germany) with 1 M NaOH (Sigma-Aldrich Co., Steinheim, Germany) in order to obtain chitosan samples with a 90% degree of deacetylation (DD). The hydrogel had a concentration of 3% and a pH of 5,6. Our biomaterial was kept within the surgical extraction site through primary closure of the wound.

### 2.2. Definition of the Variables in Study and Follow-Up of Patients

**Figure 3:**
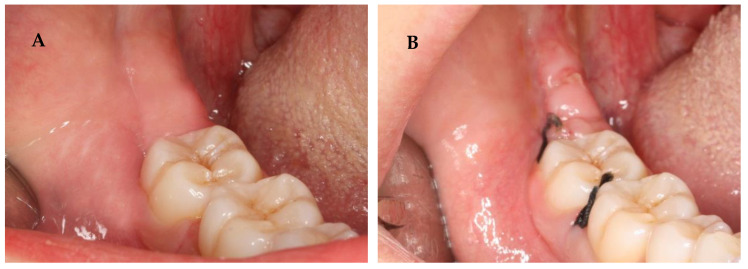
In these images, we can observe the preoperative (**A**) and postoperative (**B**) redness.

**Figure 4:**
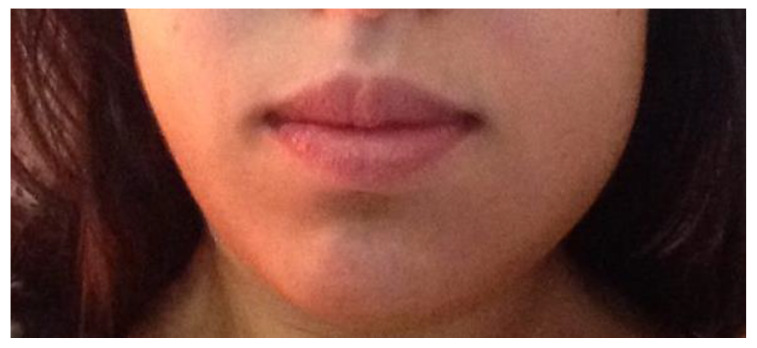
In this image, the postoperative swelling is visible on the left side of the face of the patient after 3.8 teeth extraction.

Following surgery, all patients were prescribed an antibiotic, analgesic, and NSAID (amoxicillin and clavulanic acid, paracetamol, and Ibuprofen) [42,43]. Corticosteroid (Deflazacort) was prescribed only if osteotomy was performed. One week after the procedure, the sutures were removed. The healing process was assessed by an analysis of inflammation signs (redness, swelling, pain, and loss of function) and bleeding from the post-extraction sockets. These factors were subjected to a semi-quantitative analysis. Notably, the evaluation of these variables is purely subjective. The initial color of the oral mucosa (Figure 3) and the shape of the face/mandibular angle (Figure 4) were examined to evaluate preoperative and postoperative redness and swelling, respectively. Loss of function was assessed by the presence or absence of trismus. A questionnaire was also conducted to monitor the postoperative symptoms and medication. In turn, pain intensity was evaluated through a Visual Analogue Scale (VAS) [44]. 

Patients were followed up 24 h, 48 h, 72 h, and 1 and 2 weeks after tooth extraction. At each post-surgery visit, the healing process was photographed for subsequent analysis.

## 3. Results

**Figure 5:**
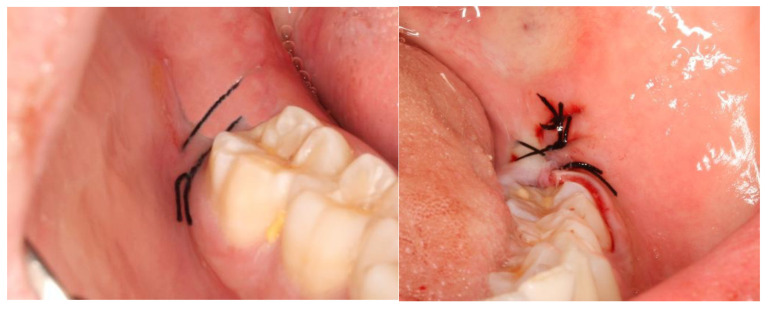
A case example of a patient’s follow-up, 24 h post-surgery. On the left, the result of tooth extraction where chitosan was applied to the socket; on the right, the side subjected to surgery without chitosan application.

**Figure 6:**
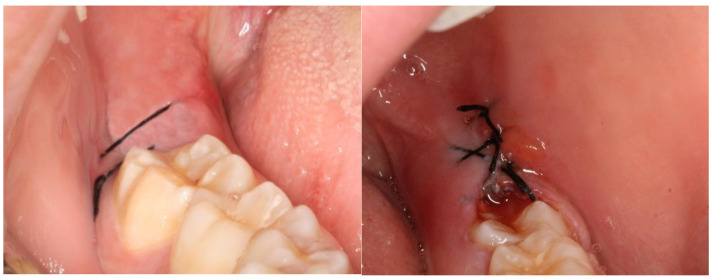
A case example of a patient’s follow-up, 48 h post-surgery. On the left, the result of tooth extraction where chitosan was applied to the socket; on the right, the side subjected to surgery without chitosan application.

**Figure 7:**
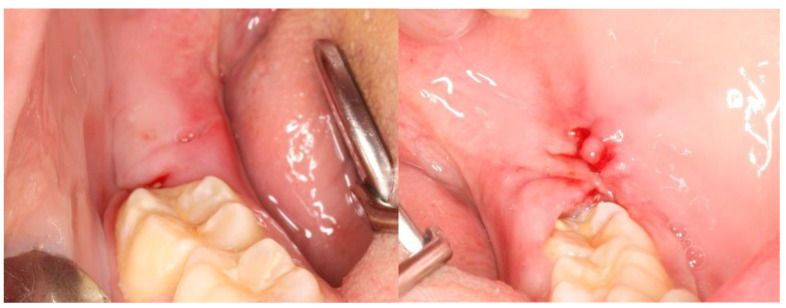
A case example of a patient’s follow-up, 72 h post-surgery. On the left, the result of tooth extraction where chitosan was applied to the socket; on the right, the side subjected to surgery with chitosan application.

**Figure 8:**
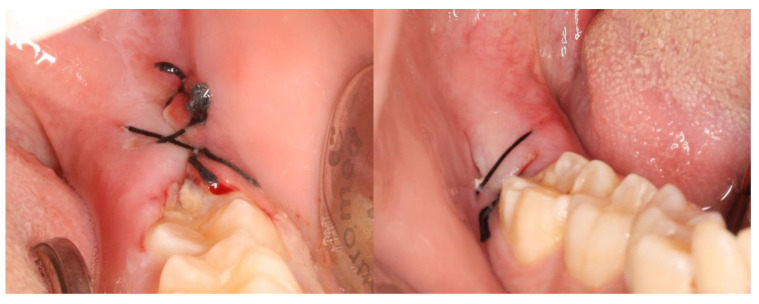
A case example of a patient’s follow-up, 1 week after surgery. On the left, the result of tooth extraction where chitosan was applied to the socket; on the right, the side subjected to surgery with chitosan application.

**Figure 9:**
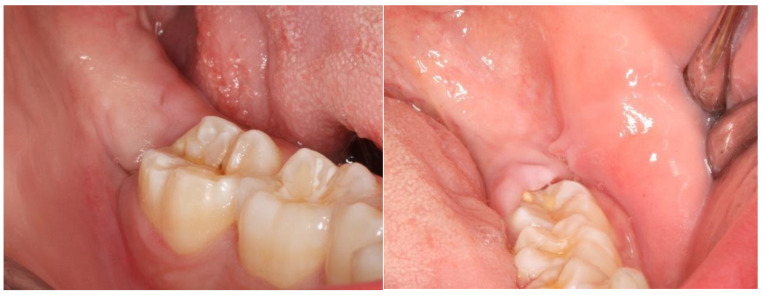
A case example of a patient’s follow-up, 2 weeks after surgery. On the left, the result of tooth extraction where chitosan was applied to the socket; on the right, the side subjected to surgery with chitosan application.

This split-mouth study included eight patients (62.5% female and 37.5% male) aged between 19 and 27 years (mean age: 22 years). The wound healing process was assessed based on a variable set, which included the evaluation of redness, pain, swelling, loss of function, and bleeding (Table S2). The photographic records of the intervened areas are shown below, where the effects of chitosan are visible, with significant differences between the control group and the experimental group (Figure 5, Figure 6, Figure 7, Figure 8 and Figure 9).

**Figure 10:**
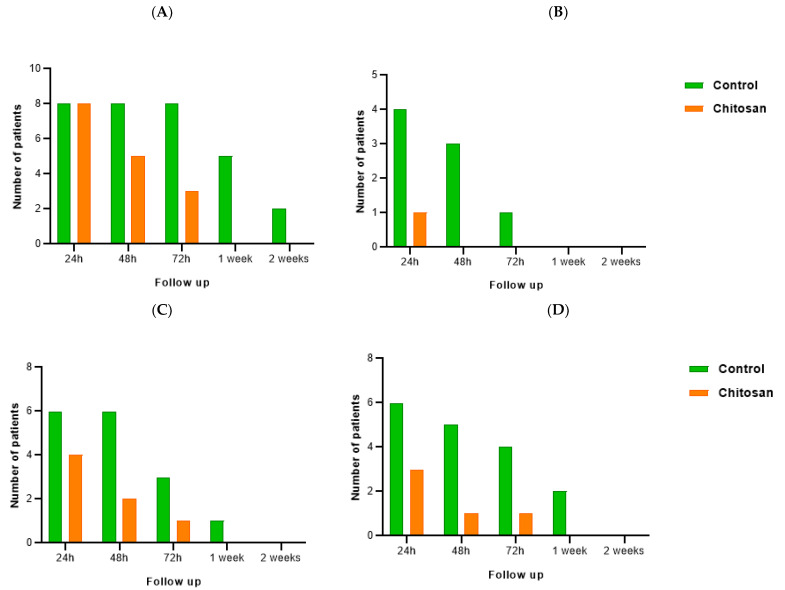
Follow-up of inflammation signs of patients: redness (**A**), pain (**B**), swelling (**C**), and loss of function (**D**) from the control group (non-chitosan sockets) and the experimental group (subjected to the application of chitosan). Redness was examined through the color of the oral mucosa; pain intensity was recorded using VAS; the shape of the face/mandibular angles was inspected for swelling evaluation; and loss of function was determined if the patient had restriction of the range of motion of the jaws.

The effects of chitosan on the inflammatory process (Figure 10) suggest they are significant. Regarding redness (Figure 10A), on the 3rd day of follow-up, all patients who had tooth extractions without chitosan administered (control) demonstrated signs of redness. After one week of the intervention, it was observed in six patients, and at the last follow-up visit (2 weeks), only two people showed redness. On the other hand, after 72 h, no redness was observed at the extraction site where chitosan was applied. Indeed, after 48 h, chitosan demonstrated a beneficial impact on this inflammatory sign, with a considerable reduction in redness in the chitosan socket compared to the control group. Pain intensity was recorded in the VAS. Only one patient recalled having discomfort in the first 24 h following surgery in the chitosan socket (Figure 10B), whereas in the cases where the biomaterial was not applied, four patients (50%) experienced pain in the first 24 h and at least one patient continued to register pain until the third day after the intervention. The difference in face shape and mandibular angles was noticeable in most patients (Figure 10C): in the control site, six patients had swelling up to 48 h after tooth extraction, and by the third visit (72 h), it was still observed in three patients; only by the second week, swelling was not evident in all patients. Nevertheless, despite being visible at the site treated with chitosan, a reduction in the number of patients with swelling was observed, and full recovery occurred earlier (in the first week). Finally, the inflammatory process was also evaluated based on loss of function (Figure 10D), particularly the presence of trismus. Chitosan’s positive benefits are once again evident. In comparison to the control group, where most patients had mandibular restriction for up to 72 h following the procedure, the number of individuals registering loss of function at the site where the biopolymer was applied was negligible: only three people demonstrated trismus after 24 h, and only one evidenced trismus after 48 h and 72 h. 

**Figure 11:**
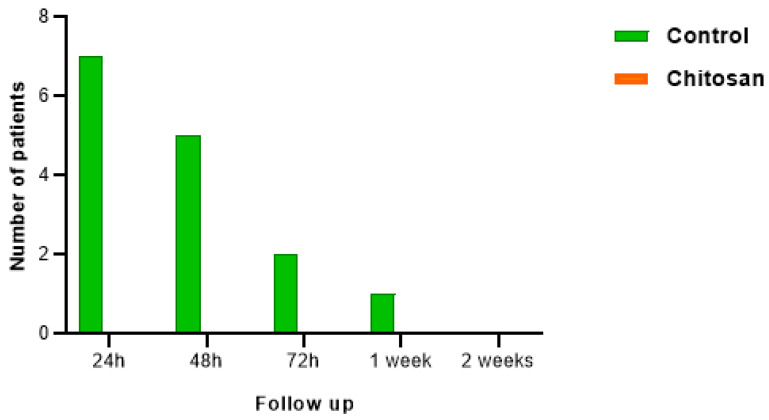
Analysis of bleeding in patients after tooth extraction in the control group and the experimental (chitosan) group.

The effects of chitosan hydrogel on bleeding were even more pronounced (Figure 11). While in sockets without chitosan, bleeding was recorded, no bleeding was observed in any socket with biopolymer following tooth extraction.

## 4. Discussion

The ideal biomaterial for wound healing must fulfill certain requirements: it must be biocompatible, ensure that the wound remains moist with exudates but not macerated, be free of infection, guarantee uniform cell distribution, maintain cell viability and phenotype, and induce the migration and proliferation of epithelial cells, fibroblasts, and endothelial cells, as well as the synthesis of extracellular matrix components for wound repair [6]. 

Chitosan is a linear copolymer derived from crustaceans’ exoskeletons, such as shrimp and crab. It has been largely investigated due to its beneficial properties in medical areas. Its chemical properties (its solubility in acidic environments), as well as its biocompatibility, biodegradability, hemostasis, and antibacterial properties make chitosan hydrogel a highly desirable product [8,24,45], particularly in the wound healing process.

According to the redness data analysis (Figure 10), all patients showed redness in the surgical area 24 h after each surgery. The differences begin to emerge in the next days of follow-up. In the control cases, all patients exhibited redness at 48 h and 72 h. One week after surgery, the percentage of patients with redness decreased to 62.5% and in the second week to 25%. In surgeries where chitosan was applied, a progressive decrease in the percentage of patients with redness was observed: after 48 h and 72 h, the percentage was 62.5% and 37.5%, respectively. The surgery region showed no redness a week later. After 2 weeks of follow up, the visual result of healing of the oral tissue was similar in both cases. These findings can be explained by chitosan’s capacity to actively participate in the early phase of wound healing and increase angiogenesis. In other words, chitosan attracts and activates neutrophils and macrophages and enhances the expression of cytokines and growth factors [7,24], as well as promoting wound healing by stimulating granulation tissue formation or re-epithelialization [7].

Regarding pain intensity, the VAS scale [44,46] used in the patients’ follow-up allows us to infer that only one patient had pain the day after surgery when chitosan was used. On other hand, four patients (50% of our sample) had the same symptom 24 h after surgery. The analgesic effect of chitosan may be attributed to its capacity to absorb bradykinin [47]. The presence of acetic acid until the complete breakdown of chitosan in oral fluids may explain a possible initial slightly raised pain score at chitosan sites [37].

In terms of swelling and loss of function, we also verified a slight decrease in cases where chitosan was used. It should be noted that swelling and loss of function, in most cases, corresponded to cases in which osteotomy had been performed, despite being minimally invasive. Following lower third molar surgery, it was observed that longer surgical interventions caused more pain, swelling, and loss of function [48]. 

The hydrophilic surface of chitosan enhances cell adhesion and proliferation [49]. Platelets adhere and aggregate on chitosan, promoting hemostasis. This supports previous findings that chitosan reduces the inflammatory response, therefore improving the inflammation stage. As a result, the greater proliferation and excellent collagen deposition during remodeling accelerate the wound healing process [50]. This biopolymer improves the wound tensile strength by speeding up the fibroblastic synthesis of collagen in the first few days of wound healing [51]. Because of its bioadhesive property, chitosan is expected to remain on the application site for a prolonged time [52].

In vivo and in vitro studies have indicated the pro-inflammatory effect of chitosan. When chitosan oligomers enzymatic degradation occurs in a wound environment, macrophages are stimulated and the migratory activity is significantly increased [24].

In vitro assays showed that chitosan can stimulate the proliferation of human periodontal ligament cells and recruit vascular tissue growth, which provides considerable evidence that chitosan helps the regeneration of periodontal cells [20]. Silva et al. [18] assessed whether chitosan is able to stimulate cell proliferation and if it collaborates with other growth factors such as PDGF to exert its mitogenic effect. They observed that chitosan stimulates cell viability and promotes cell proliferation in human gingival fibroblasts. Moreover, periodontal ligament in the presence of chitosan presented a more regular pattern and a denser fiber arrangement than those in the surgical control group [20].

Histological observations in wound-healing mouse model experiments showed that wounds treated with chitosan hydrogel have advanced granulation tissue formation and epithelialization when compared to the control. The application of this hydrogel onto an open wound induces significant wound contraction and accelerates its closure and healing [53].

The role of chitosan in tissue regeneration has been supported by several studies. The amino groups of this compound are recognized by the immune system, inducing inflammatory cells and fibroblasts to migrate to the wound region and activating them to produce multiple cytokines. Fibroblasts are stimulated by chitosan molecules to secrete IL-8 and other cytokines, which, in turn, could induce angiogenesis, fibrosis, and epithelialization [28,53]. Boynueǧri et al. [52] evaluated the effect of chitosan gel, comparing its combination with collagen membrane to flap alone on periodontal regeneration applied into the intraosseous lesions. They concluded that all treatment modalities provide improvements in clinical measurements of tissue regeneration. The application of chitosan in gel form did not trigger an inflammatory reaction. 

The antimicrobial activity of chitosan also promotes the repair of damaged tissue, preventing infection of the wound [7,50,51]. This biopolymer increases the permeability of the inner and outer membranes and ultimately disrupts the bacterial cell membranes, releasing their contents. It provides an antibacterial barrier against a wide range of Gram-positive and Gram-negative organisms, including methicillin-resistant Staphylococcus, mancomycin-resistant Enterococcus, and *Acinetobacter baumannii* [39].

Chitosan also has the ability to impair the colonization of the tooth surface by *S. mutans*, which prevents possible infection after surgery [52]. Xu et al. [20] studied chitosan membranes in animal models, and they concluded that they are cheaper and, due to their property of bacteriostasis, may reduce bacteria contamination and benefit periodontal tissue regeneration. 

Hemostatic activity is an important phase in the early treatment of an injury [7]. In our experimental study (Figure 10), in the control cases, seven out of the eight patients (87.5%) bled the day after surgery, five patients (62.5%) continued to bleed past 48 h, two patients (25%) bled at 72 h, and one patient (12.5%) was still bleeding one week after surgery. This was not observed in the experimental cases. None of these patients had bleeding in the days following surgery, allowing us to report a peculiar ability of chitosan in hemostasis. 

This can be explained by the interaction of chitosan with blood cells. The outer membranes of erythrocytes and platelets are negatively charged and are attracted to the positively charged reactive amino groups of chitosan, leading to platelet activation and thrombus formation [29,40]. Okamoto et al. [54] corroborate this theory. These authors concluded that chitin and chitosan both enhance blood coagulation, with chitosan being more effective. Their findings demonstrated that chitosan reduces blood clotting time, due to its physical effect and chemical structure; notably, amino groups were significant in platelet aggregation. In addition, electrophoretic and Western blot analysis of red blood cell surface proteins revealed that chitosan microfibers were bound to band three of red blood cells and, therefore, this interaction leads to the activation of the intrinsic coagulation cascade [55].

Patients on oral anticoagulant treatment generally require additional care in the field of dentistry. The common, and recommended, approach is to suspend medication for 3 to 4 days prior to surgery. Nevertheless, patients are more exposed to a higher risk of thromboembolism, myocardial infarction, and cardiovascular accidents. Therefore, the use of chitosan can minimize this risk, allowing the patient to continue their antiplatelet medication, despite the need for postoperative evaluation and management of the patient’s International Normalized Ratio (INR) status [39].

HemCon®, an FDA-approved dressing made from chitosan, is molded to form a highly electropositive sponge-like material that binds to negatively-charged red blood cells. Consequently, it rapidly forms a viscous clot, sealing the wound site and facilitating hemostasis. The use of this product in extraction sites (including patients taking oral anticoagulant therapy) was evaluated, and hemostasis was achieved in less than 1 min, the control wounds taking 9.54 min [37,56]. Kale et al. [39] compared the effectiveness of this product and conventional methods in the post-extraction bleeding management in patients undergoing oral antiplatelet therapy. The outcomes demonstrated that time to hemostasis was shorter in the case of the chitosan product. According to the authors, the self-adhesive nature of HemCon® is caused by the electrostatic attraction of red blood cells to chitosan, promoting the formation of a dense viscous mass that provides adhesion and sealing of the wound site. It allows the body to spontaneously produce a clot and effectively activate its coagulation pathway, initially forming organized platelets [39]. Furthermore, it was found to cause an improvement in postoperative healing with minimal complications compared to the control site. This may be attributed to a release of growth factor from human platelets stimulated by chitosan exposure and the antibacterial properties of chitosan. The investigation involved the application of a HemCon® in a full-thickness excisional wound in mice that had been infected with pathogenic bacteria such as Pseudomonas aeruginosa, Proteus mirabilis, and Staphylococcus aureus, and showed that chitosan not only kills the bacteria but also stimulates wound healing [57].

Aktop et al. [41] investigated the hemostatic effects of other chitosan-based products in warfarin-treated rats and observed great success in controlling bleeding intraoperatively and postoperatively. It interacts directly with red blood cells and platelets to establish a cross-linked barrier clot, independent of native factors. Additionally, it boosts the tissue factor activity, the major initiator of the extrinsic coagulation cascade that is involved in all phases of the host response to wounding, implying a likely central role for tissue factor in wound healing. Insufficient resorption of chitosan granules has also been documented. For this reason, for successful early-stage wound healing and to avoid epithelization problems, the authors recommend carefully and totally removing them from the wound.

Other studies involving other chitosan abilities, such as osteogenic activity, have evidenced that tissue and cell formation during wound healing augmented new bone growth and had significant hemostatic properties [58]. Furthermore, chitosan induces the development of more new cementum and bone, with densely arranged cementoblasts and osteoblasts along the new bone surface [20].

From an anti-inflammatory and regenerative perspective, the mechanisms unveiled by recent in vitro studies have shown that chitosan impacts the cyclooxygenase pathway, as chitosan-containing gel has been shown to significantly decrease the PGE2 production in IL 1β-treated cells [59]. Likewise, in addition to the downregulation of the COX pathway, chitosan also appears to downregulate JNK activity in gingival fibroblasts, making it one of the intracellular targets modulated by chitosan particles in gingival fibroblasts exposed to inflammatory stimuli such as IL-1β [60]. Moreover, the presence of connective tissue ingrowth and mineralization within cell-seeded scaffolds in the bone region has been reported. Mineralization within the scaffold pores has been confirmed by osteopontin and sialoprotein bone markers, with scaffolds seeded with human osteoblasts depicting the high level of osteoprotegerin, while those seeded with human periodontal ligament fibroblasts were associated with high levels of RANKL, a known potent inducer of osteoclast differentiation and activation [61]. 

Overall, despite its limitations, the results of this observational feasibility split-mouth study allow us to conclude that chitosan is likely to bear a positive influence on the healing process. In fact, although some classic signs of inflammation were observed in a short period of time where chitosan acts, overall, compared to the control, the beneficial effect of chitosan is far from negligible. 

The unique biologic properties and the inexpensive cost of chitosan are important for the practice in dentistry since they eliminate the requirement for additional material, such as barrier membranes and grafts in regenerative dentistry. This means shorter procedures and a more cost-effective treatment option for the patient [36,52]. Moreover, it could be important in the postoperative quality of patients, allowing rapid healing of the wound with less complications. 

## 5. Future Perspectives

In this study, a semi-quantitative evaluation of the variable in study was performed. It was ensured to be a blinded study, although the authors were aware of which side the chitosan was applied. 

Regrettably, we recognize the sample size as one of the major weaknesses of this pilot study. Some conclusions can already be drawn, meaning further validation should cover a larger cohort of patients. The age range is also considerably short. Our patients were young, aged between 19 and 27 years old. This issue must also be overcome.

Additionally, future studies may also consider not only the influence of chitosan in soft tissues but also in the regeneration of hard tissues. In a further analysis, it would be important to analyze the data more specifically using, for example, biomarkers of inflammation. 

Moreover, the qualitative approach that authors used as a surrogate of the inflammatory proprieties of the chitosan needs to be complemented by laboratory tests aiming to find the underlying physiological aspects. Hence, future studies will benefit from adding laboratory experiments aiming to assess aspects such as inflammatory, adhesion biomarker, and cell immune patterns (platelets and macrophages) by flow cytometry or other techniques from biopsies.

Another interesting point would be to assess if the verified hydrogel hemostatic capacity is entirely due to chitosan properties or due to the presence and action of acetic acid and whether the clinical outcomes are dose-dependent. Other studies should be conducted by associating chitosan with other drugs in order to check whether there is a possibility to influence and enhance the healing process.

## Data Availability

Not applicable.

## Supplementary Materials

The following supporting information can be downloaded at: https://www.mdpi.com/article/10.3390/nano13040706/s1, Figure S1. Patient 1: Orthopantomography. Figure S2. Patient 2: Orthopantomography. Figure S3. Patient 3: Orthopantomography. Figure S4. Patient 4: Orthopantomography. Figure S5. Patient 5: Orthopantomography. Figure S6. Patient 6: Orthopantomography. Figure S7. Patient 7: Orthopantomography. Figure S8. Patient 8: Orthopantomography. Table S1. Demographic characteristics of all patients. Table S2. Postoperative evaluation of all patients. Pain intensity, redness, swelling, loss of function, and bleeding were recorded in sockets with chitosan (Q) and without chitosan (control, C).
