# Supplementary material for: The Effects of Chitosan on the Healing Process of Oral Mucosa: An Observational Cohort Feasibility Split-Mouth Study
Source: Nanomaterials (Basel). 2023 Feb 12;13(4):706. doi: 10.3390/nano13040706 (PMC9963900; doi:10.3390/nano13040706)
Supplement: Supplementary file 1 [file nanomaterials-13-00706-s001.zip › nanomaterials-2200012-supplementary.pdf]

Supplementary Materials: The effects of the chitosan on the healing process of the oral mucosa

Clinical information about each patient's case

Patient 1

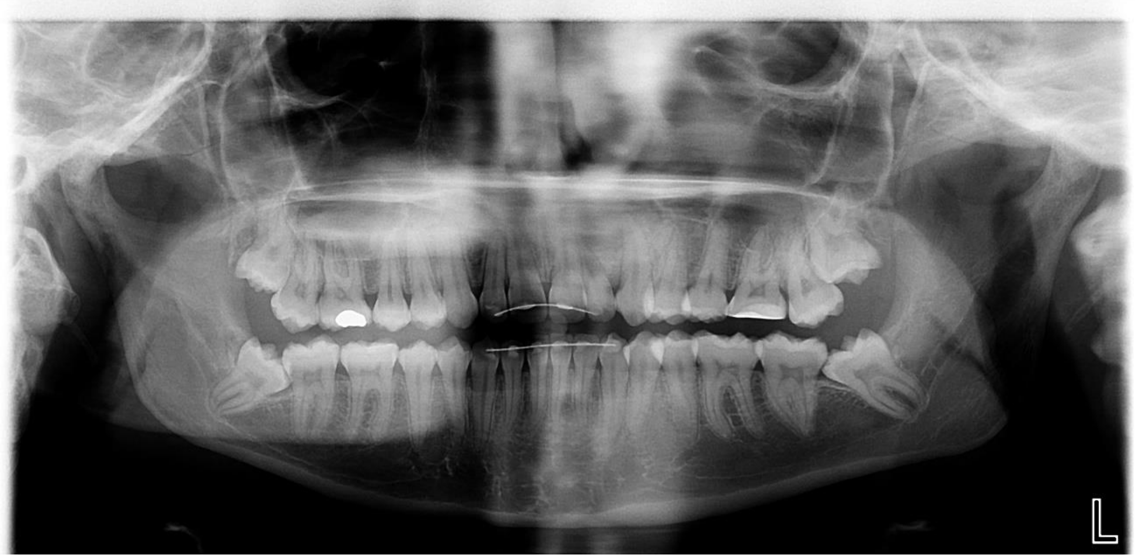

Figure S1. Patient 1: Orthopantomography.

Chitosan (Q): tooth 4.8
- Pell and Gregory classification: I B
- Intervention Time: <30 minutes
- Suture: 3 simple points

Control (C): tooth 3.8
- Pell and Gregory classification: I B
- Intervention Time: <30 minutes
- Suture: 3 simple points

Patient 2

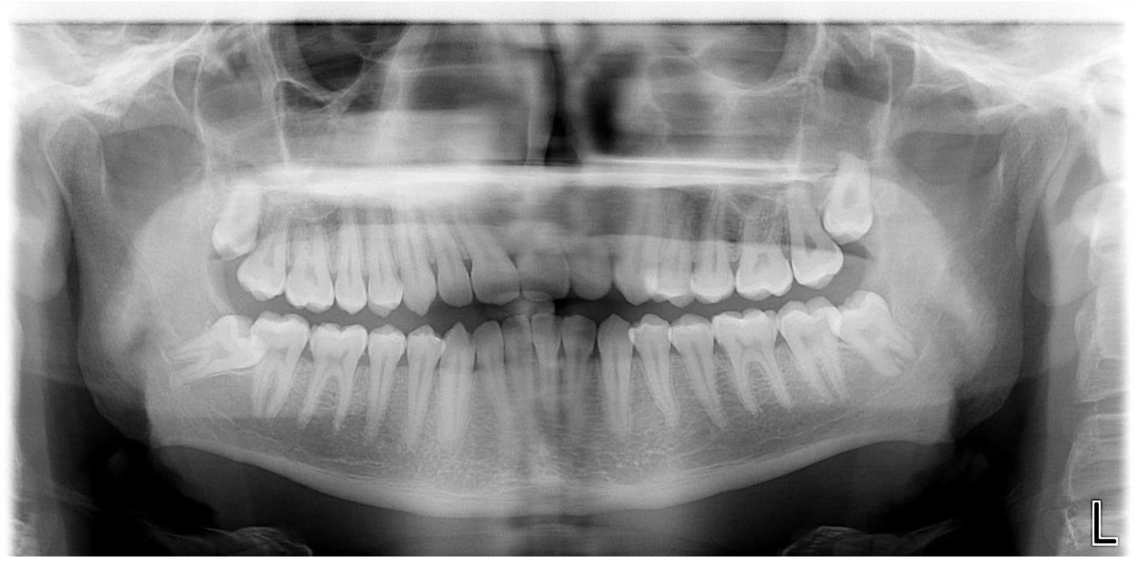

Figure S2. Patient 2: Orthopantomography.

Chitosan (Q): tooth 3.8

- Pell and Gregory classification: II B
- Intervention time: <30 minutes;
- Suture: 3 simple points.

Control (C): Tooth 4.8 - It was performed osteotomy.

- Pell and Gregory classification: II B
- Intervention time: <30 minutes;
- Suture: 3 simple points.

Patient 3

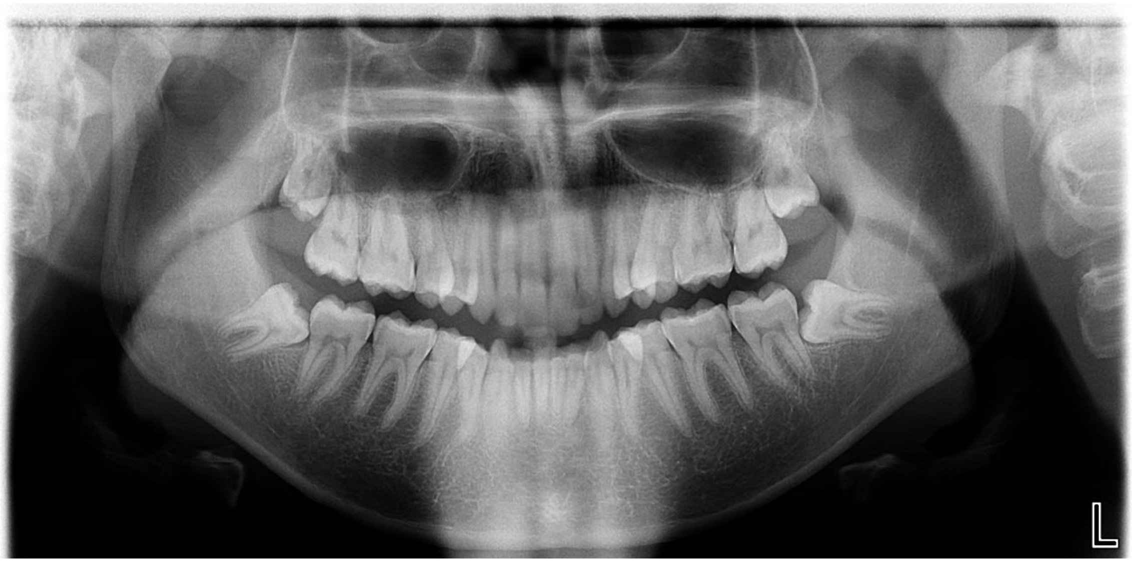

Figure S3. Patient 3: Orthopantomography.

Chitosan (Q): tooth 4.8 - It was performed osteotomy.

- Pell and Gregory classification: I B
- Intervention time: <30 minutes;
- Suture: 3 simple points.

Control (C): Tooth 3.8

- Pell and Gregory classification: II B
- Intervention Time: <30 minutes;
- Suture: 3 simple points

Patient 4

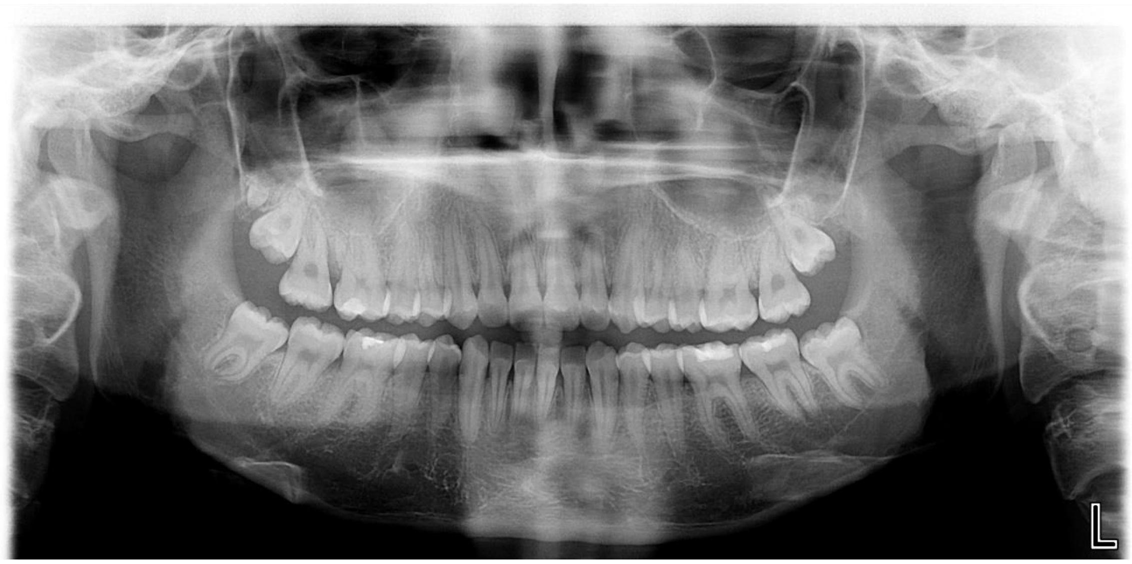

Figure S4. Patient 4: Orthopantomography.

Chitosan (Q): tooth 4.8

- Pell and Gregory classification: I B;
- Intervention Time: <30 minutes;
- Suture: 4 simple points.

Control (C): Tooth 3.8

- Pell and Gregory classification: I B;
- Intervention Time: <30 minutes;
- Suture: 3 simple points.

Patient 5

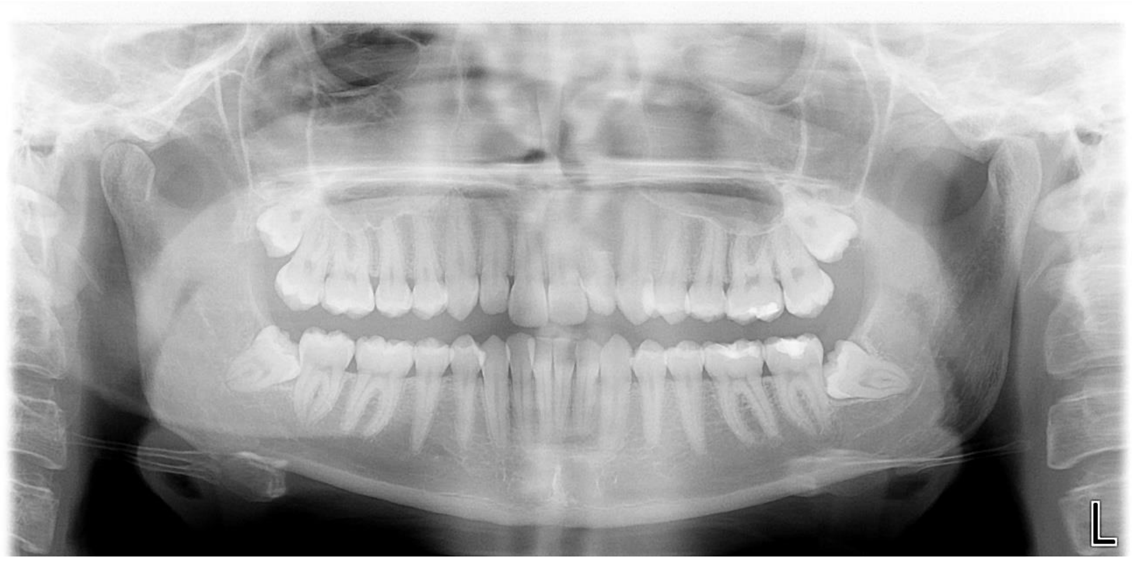

Figure S5. Patient 5: Orthopantomography.

Chitosan (Q): tooth 3.8 - It was performed osteotomy

- Pell and Gregory classification: II B;
- Intervention Time: 30 - 60 minutes.
- Suture: 3 simple points.

Control (C): tooth 4.8

- Pell and Gregory classification: I B;
- Intervention Time: <30 minutes.

- Suture: 3 simple points.

Patient 6

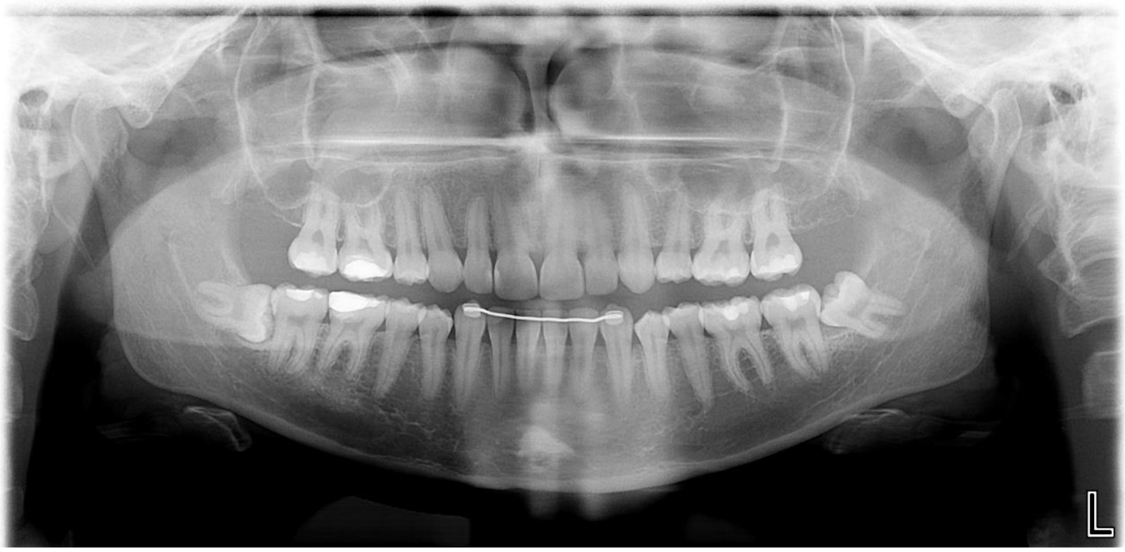

Figure S6. Patient 6: Orthopantomography.

Chitosan (Q): tooth 3.8
- Pell and Gregory classification: I B
- Intervention Time: <30 minutes;
- Suture: 3 simple points.

Control (C): tooth 4.8 - It was performed osteotomy;
- Pell and Gregory classification: II B
- Intervention Time: 30 - 60 minutes;
- Suture: 3 simple points.

Patient 7

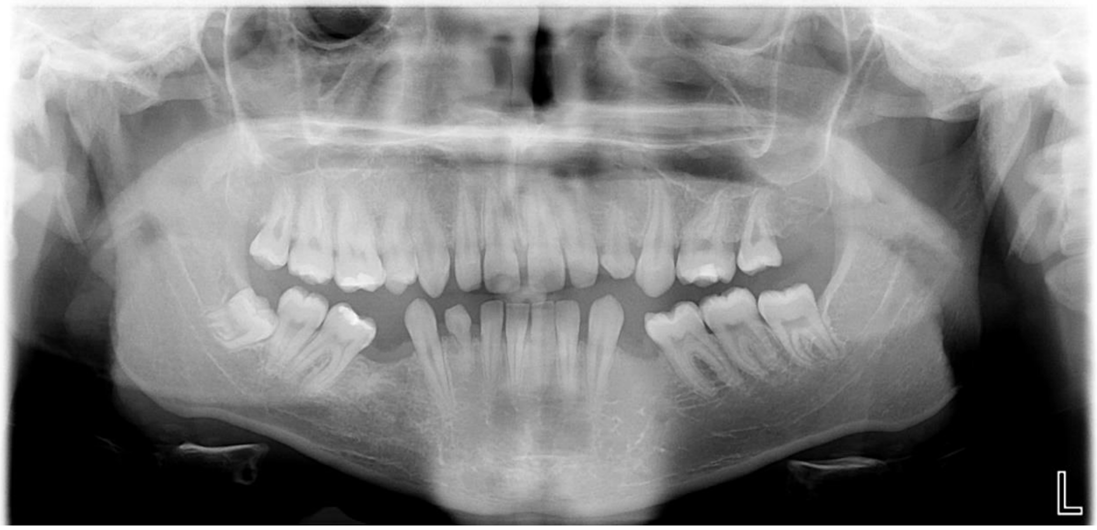

Figure S7. Patient 7: Orthopantomography.

Chitosan (Q): 4.8
- Pell and Gregory classification: II B
- Intervention Time: <30 minutes;

- Suture: 3 simple points.

Control (C): 3.8
- Pell and Gregory classification; I B;
- Intervention Time: <30 minutes;
- Suture: 3 simple points.

Patient 8

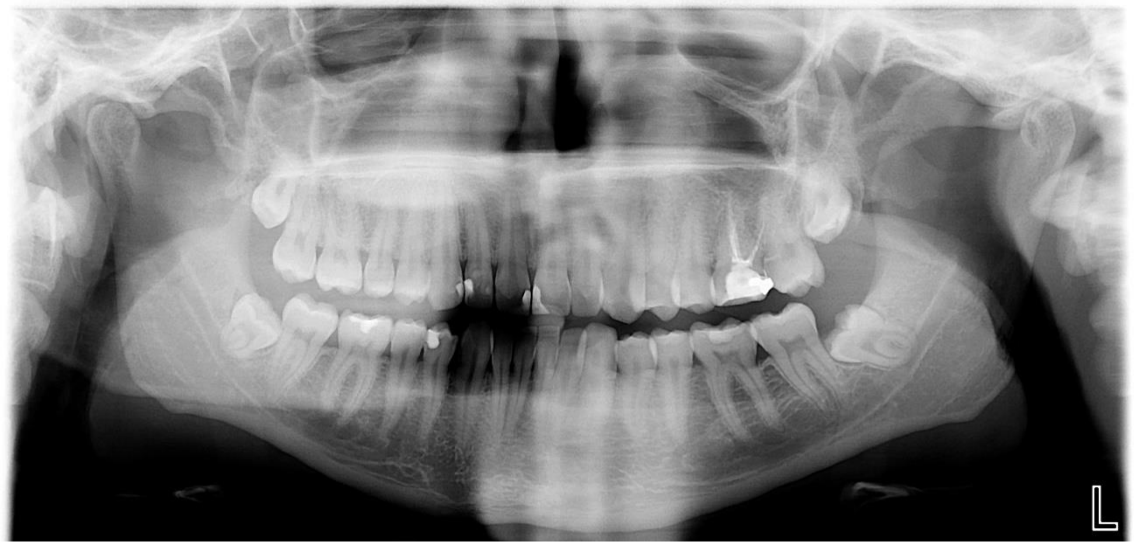

Figure S8. Patient 8: Orthopantomography.

Chitosan (Q): tooth 4.8
- Pell and Gregory classification: II B;
- Intervention Time: <30 minutes;
- Suture: 3 simple points.

Control (C): tooth 3.8
- Pell and Gregory classification: I B;
- Intervention Time: <30 minutes;
- Suture: 3 simple points.

Table S1. Demographic characteristics of all patients.

Patient	Gender	Age (years)
1	Female	21
2	Male	19
3	Male	22
4	Female	21
5	Female	21
6	Female	24
7	Male	27
8	Female	22

Follow-up of patients

Table S2. Postoperative evaluation of all patients. Pain intensity, redness, swelling, loss of function, and bleeding were recorded in sockets with chitosan (Q) and without chitosan (control, C).

Patients / Variables	Follow up	1		2		3		4		5		6		7		8	
		Q	C	Q	*C	*Q	C	Q	C	*Q	C	Q	*C	Q	C	Q	C
**Pain**	24h	-	•	-	-	-	-	-	•	•	-	-	•	-	-	-	•
	48h	-	•	-	-	-	-	-	•	-	-	-	•	-	-	-	-
	72h	-	-	-	-	-	-	-	•	-	-	-	-	-	-	-	-
	1 week	-	-	-	-	-	-	-	-	-	-	-	-	-	-	-	-
	2 weeks	-	-	-	-	-	-	-	-	-	-	-	-	-	-	-	-
**Redness**	24h	•	•	•	•	•	•	•	•	•	•	•	•	•	•	•	•
	48h	-	•	•	•	-	•	•	•	•	•	-	•	•	•	•	•
	72h	-	•	•	•	-	•	•	•	-	•	-	•	-	•	•	•
	1 week	-	-	-	•	-	•	-	•	-	-	-	-	-	•	-	•
	2 weeks	-	-	-	•	-	-	-	•	-	-	-	-	-	-	-	-
**Swelling**	24h	-	•	•	•	-	-	•	•	•	•	•	•	-	-	-	•
	48h	-	•	-	•	-	-	-	•	•	•	•	•	-	-	-	•
	72h	-	•	-	•	-	-	-	•	•	-	-	-	-	-	-	-
	1 week	-	-	-	-	-	-	-	•	-	-	-	-	-	-	-	-
	2 weeks	-	-	-	-	-	-	-	-	-	-	-	-	-	-	-	-
**Loss of function**	24h	-	•	•	•	-	-	-	•	•	•	-	•	-	-	•	•
	48h	-	•	-	•	-	-	-	•	•	-	-	•	-	-	-	•
	72h	-	•	-	-	-	-	-	•	•	-	-	•	-	-	-	•
	1 week	-	-	-	-	-	-	-	•	-	-	-	•	-	-	-	-
	2 weeks	-	-	-	-	-	-	-	-	-	-	-	-	-	-	-	-
**Bleeding**	24h	-	•	-	•	-	•	-	•	-	•	-	-	-	•	-	•
	48h	-	•	-	•	-	•	-	-	-	-	-	-	-	•	-	•
	72h	-	-	-	•	-	-	-	-	-	-	-	-	-	-	-	•
	1 week	-	-	-	-	-	-	-	-	-	-	-	-	-	-	-	•
	2 weeks	-	-	-	-	-	-	-	-	-	-	-	-	-	-	-	-
